# ATOJIN: A Natural Products Mixture, Alleviates Atopic Dermatitis in DNCB‐Induced NC/Nga Mice

**DOI:** 10.1155/mi/3444278

**Published:** 2026-03-14

**Authors:** Sang-Eun Lee, Kwang-Jin Cho, Min-Woo Kim, Hee-Sun Yim, Seong-Gyu Ko

**Affiliations:** ^1^ Department of Science in Korean Medicine, Graduate School, Kyung Hee University, Seoul, Republic of Korea, khu.ac.kr; ^2^ Institute for Research Center in K-LAB, Seoul, Republic of Korea; ^3^ Department of Preventive Medicine, College of Korean Medicine, Kyung Hee University, Seoul, Republic of Korea, khu.ac.kr

**Keywords:** 24-dinitrochlorobenzene, atopic dermatitis, natural compounds, natural products mixture, NC/Nga mice

## Abstract

Atopic dermatitis (AD) is a chronic, relapsing inflammatory skin disorder with increasing global prevalence. ATOJIN is a natural product composed of *Phellodendron amurense Ruprecht* (PAR), *Schizonepeta tenuifolia* (ST), *Sophora flavescens* (SF), *Glycyrrhiza uralensis* (GU), and *Liriope platyphylla* (LP), all known for their anti‐inflammatory properties. This study aims to evaluate the therapeutic potential of ATOJIN in a 2,4‐dinitrochlorobenzene (DNCB)‐induced AD mouse model by assessing its effects on inflammation and immune regulation. The therapeutic efficacy of ATOJIN was evaluated through the analysis of white blood cell subtypes, serum immunoglobulin E (IgE) levels, pro‐inflammatory cytokines, and histological assessments of inflammatory and mast cell infiltration, focusing on systemic immune modulation. ATOJIN effectively alleviated AD lesions and symptoms in DNCB‐induced mice, demonstrating significant improvements in dermatitis scores, ear thickness, and spleen weight. It also reduced epidermal thickness and the infiltration of inflammatory and mast cells. Furthermore, ATOJIN modulated serum levels of IgE and pro‐inflammatory cytokines, including interleukin (IL)−2, IL‐4, IL‐6, and tumor necrosis factor (TNF)‐α, indicating systemic anti‐inflammatory effects. These results suggest that ATOJIN mitigates AD symptoms by modulating inflammatory responses, and its efficacy, comparable to or even superior to that of the topical standard‐of‐care tacrolimus, highlights its potential as a promising standalone oral therapeutic agent for the systemic management of AD.

## 1. Introduction

Atopic dermatitis (AD) is a chronic inflammatory skin disorder that affects ~20% of children worldwide. It typically manifests during infancy or childhood, although it can also occur in adults. The prevalence of AD has steadily increased over the years, with an estimated 15%–20% of the global population currently affected [1]. The pathogenesis of AD is not fully understood; however, several factors contribute to its development, including allergens, microbial exposure, immune dysfunction, and damage to the skin barrier [2]. This condition is characterized by elevated levels of inflammatory cytokines and immunoglobulin E (IgE) in the blood, presenting a range of symptoms including erythema, itchy rashes, dryness, and skin lesions [3]. It is also linked to other allergic conditions, such as asthma and rhinitis, which require ongoing management [4].

Systemic immunomodulation is necessary to manage recurrent or moderate‐to‐severe symptoms of AD. Although steroid treatment is commonly recommended for AD, its long‐term use presents challenges due to side effects such as steroid rosacea, acne, atrophy, perioral dermatitis, and telangiectasia [4]. Topical immunosuppressants, such as tacrolimus, have been introduced as steroid‐free alternative anti‐inflammatory treatments for patients with AD, providing a safer and more effective option [5]. However, several side effects associated with tacrolimus have been reported, including pruritus, burning sensations on the skin, flu‐like symptoms, headaches, coughing, and ocular burning [6]. These side effects restrict the use of systemic therapies in infants and young children [7, 8]. Consequently, there is a critical need for new treatments that utilize natural ingredients, which are both effective and minimize side effects, especially since most patients with AD are infants or young children [9–11]. In this context, natural products have gained attention as alternative treatments for AD, with certain herbal medicines traditionally used to manage the condition [12–14].

ATOJIN is a mixture of natural product extracts from *Phellodendron amurense Ruprecht* (PAR) [13, 15], *Schizonepeta tenuifolia* (ST) [16–18], *Sophora flavescens* (SF) [19–21], *Glycyrrhiza uralensis* (GU) [22, 23], and *Liriope platyphylla* (LP) [24, 25]. Each component exerts anti‐inflammatory effects by scavenging free radicals and inhibiting inflammation. We previously evaluated the efficacy and mechanisms of the topical formulation, conducting both qualitative and quantitative analyses of its components [26]. This study further investigates and characterizes its components to identify additional active constituents. While topical formulations such as tacrolimus are effective for localized treatment, they have limited capacity to modulate systemic immune responses and may be inadequate for managing extensive or severe AD. To address these limitations and develop a systemic therapeutic alternative, our research on ATOJIN focuses on oral administration. This approach aims to identify its active compounds and evaluate its therapeutic efficacy in a moderate AD model, emphasizing the assessment of its overall anti‐inflammatory potential through systemic administration.

In this study, PAR, ST, SF, GU, and LP were individually extracted using 70% ethanol, and the standard compounds were identified and quantified through high‐performance liquid chromatography (HPLC) analysis. We evaluated the therapeutic efficacy of orally administered ATOJIN in treating 2,4‐dinitrochlorobenzene (DNCB)‐induced AD‐like skin lesions in the NC/Nga mouse model and compared its ability to induce a systemic immunosuppressive response with that of tacrolimus.

## 2. Methods

### 2.1. Reagents

HPLC‐grade water, acetonitrile, and methanol were obtained from JT Baker (Radnor, PA, USA), while formic acid was sourced from DAEJUNG (Siheung, Korea). Berberine, palmatine, menthone, kurarinone, glycyrrhizin, and ophiopogonin D were purchased from Sigma–Aldrich (Saint Louis, MO, USA). Pulegone and liquiritin were acquired from Supelco (Bellefonte, PA, USA), oxymatrine and matrine from EDQM (Strasbourg, France), and spicatoside A from ChemNorm (Shanghai, China). PMA and ionomycin were also purchased from Sigma–Aldrich (Saint Louis, MO, USA).

### 2.2. Plant Materials

The components of ATOJIN included PAR (Rutaceae), ST (Lamiaceae), SF (Fabaceae), GU (Fabaceae), and LP (Asparagaceae). PAR, SF, and GU were sourced from China and purchased from Enteph Herb Co., Ltd. (Daejeon, Korea). ST was also sourced from China and obtained from CK Pharm Co., Ltd. (Seoul, Korea), while LP was sourced in Korea and purchased from Sunil Herbal Medicine Co., Ltd. (Daejeon, Korea). All plant materials were processed and managed in facilities certified for good manufacturing practices.

### 2.3. Preparation of ATOJIN

ATOJIN is a mixture of individual plant extracts derived from five medicinal plants, each at a ratio of 1:1:1:1:1. The extraction of each plant raw material was outsourced to FORUSGEN (Chungju, Korea). For each plant, 100 g of dried raw material was extracted with 1 L of 70% (v/v) ethanol at 95°C for 6 h. After the initial extraction, the extract was filtered, and the residual plant material underwent a second extraction under the same conditions using fresh solvent. The filtrates from both extractions were then combined. The resulting extract, with a total volume of ~1.9 L, was concentrated under reduced pressure at 60–75°C to a final volume of 200 mL. The concentrated extract was subsequently freeze‐dried to obtain a powdered form. Each freeze‐dried plant extract was stored at −20°C until further use. Detailed extraction conditions are summarized in Table 1.

**Table 1:** Extract condition for PAR, ST, SF, GU, and LP.

Condition	70% EtOH
Herbal medicine using weight	100 g
Solvent using	10 v/v
Extract temperature (°C)	95
Concentrate condition	Rotary evaporator system (60–75°C)
Dry method	Freeze dry
Extract times	2

ATOJIN was prepared by mixing the freeze‐dried powders of five individual plant extracts in a 1:1:1:1:1 (w/w) ratio. For in vivo administration, the ATOJIN mixture was dissolved in saline just before use.

### 2.4. Liquid Chromatography–Mass Spectrometry Analysis

Samples of PAR, ST, SF, GU, and LP extracts were prepared for analysis by dissolving 10 mg of each in 1.0 mL of methanol, followed by sonication for 30 min and filtration through a 0.5 μm PTFE syringe filter. The HPLC conditions were optimized for each extract. LC–MS analysis was conducted using an Agilent 6410 Triple Quadrupole HPLC system (Agilent Technologies, Santa Clara, CA, USA) equipped with a PDA detector and three columns: Poroshell 120 EC‐C18 (2.7 μm), Extent C18 (1.8 μm) (Agilent Technologies, Santa Clara, CA, USA), and Daicel Dcpak PTZ (3 μm, Daicel Corporation, Tokyo, Japan). The mobile phase consisted of solvent A (deionized water with 0.1% formic acid) and solvent B (acetonitrile with 0.1% formic acid), with a gradient adjusted for each analyte. Samples were injected at 5.0 μL, with a mobile phase flow rate of 0.2–0.3 mL/min, maintaining the column at 40°C and the detector wavelength at 248 nm. Each extract and standard compound was analyzed under the conditions specified in Table 2.

**Table 2:** HPLC analysis of PAR, ST, SF, GU, and LP.

Material	PAR	ST	SF	GU	LP
Standard	Berberine, palmatine	Pulegone	Kurarinone, oxymatrine, matrine	Glycyrrhizin, liquritin	Menthone, pphiopagonin D, spicatoside A
Column	Poroshell 120 EC‐C18			Pcpak PTZ	Extent C18
Mobile phase	A: 0.1% FA in DW, B: 0.1% FA in Acetonitrile				
Column temp	40°C				
Flow rate	0.2 mL/min	0.2 mL/min	0.2 mL/min	0.3 mL/min	0.3 mL/min

### 2.5. Cell Viability Assay

Human dermal fibroblasts (HDFα) and the human mast cell line‐1 (HMC‐1) were purchased from the American Type Culture Collection (Manassas, VA, USA). The cells were maintained in Dulbecco’s Modified Eagle’s Medium (WelGENE, Gyeongsan, Korea) supplemented with 10% inactivated fetal bovine serum (JR Scientific, Woodland, CA, USA) and 1% penicillin/streptomycin solution (WelGENE, Gyeongsan, Korea). Cells at passage 5 were used for subsequent experiments. To measure cell viability, drug‐treated cells were incubated for 2 h after the addition of Water‐Soluble Tetrazolium 1 (WST‐1) solution. The absorbance was measured at 450 nm using an ELISA reader (Molecular Devices, San Jose, CA, USA).

### 2.6. Animals

NC/Nga (male, 7 weeks old, 20–25 g) mice were purchased from Central Lab Animal Inc. (Seoul, Korea). All mice were given ad libitum access to food and water and were housed in suitable isolated cages under pathogen‐free conditions, with a 12‐h light/12‐h dark cycle at room temperature (22–25°C). The mice were allowed to acclimate to their environment for 1 week after arrival. Animal experimental procedures were conducted in accordance with the protocols approved by the Institutional Animal Care and Use Committees of Kyung Hee University (Number KHSASP‐23‐458), in compliance with the university’s guidelines for the care and use of animals.

### 2.7. RNA Isolation and Real‐Time PCR (RT–PCR) Analysis

Cells and mouse tissues were harvested, and RNA was isolated using the easy‐BLUE Total RNA Extraction Kit (iNtRON Biotech, Seongnam, Korea) following the manufacturer’s instructions. The RNA concentration was measured using a NanoDrop ND‐1000 spectrophotometer (NanoDrop Technologies Inc., Wilmington, DE, USA). cDNA was synthesized from 2 µg of total RNA using a cDNA Synthesis Kit (TaKaRa, Otsu, Japan). RT–PCR was performed in triplicate on a Light Cycler 96 instrument (Roche, Basel, Switzerland) using the SensiFAST SYBR Hi‐ROX kit (Meridian Bioscience, Newtown, OH, USA) with gene‐specific primers (Table 3).

**Table 3:** PCR primer sequences.

Primer type	Primer target	Primer sequence
Mouse	IL‐2	F: 5′‐GCA GCT GTT GAT GGA CCT AC‐3′ R: 5′‐TCC ACC ACA GTT GCT GAC TC‐3′
	IL‐4	F: 5′‐TCG GCA TTT TGA ACG AGG TC‐3′ R: 5′‐GAA AAG CCC GAA AGA GTC TC‐3′
	IL‐6	F: 5′‐CAA GAG ACT TCC ATC CAG TTG C‐3′ R: 5′‐TTG CCG AGT TCT CAA AGT GAC‐3′
	IL‐13	F: 5′‐CGG CAG CAT GGT ATG GAG TG‐3′ R: 5′‐ATT GCA ATT GGA GAT GTT GGT CA‐3′
	TNF‐α	F: 5′‐ATG AGC ACA GAA AGC ATG ATC‐3′ R: 5′‐TAC AGG CTT GTC ACT GGA ATT‐3′
	GAPDH	F: 5′‐GAG GGG CCA TCC ACA GTC TTC‐3′ R: 5′‐CAT CAC CAT CTT CCA GGA GCG‐3′
Human	IL‐2	F: 5′‐CTG AGC AGG ATG GAG AAT TAC‐3′ R: 5′‐TGG GGG TTG GAA GAT GCT TTG ‐3′
	IL‐4	F: 5′‐TGC CTC CAA GAA CAC AAC TG‐3′ R: 5′‐CTC TGG TTG GCT TCC TTC AC‐3′
	IL‐6	F: 5′‐AAC CTT CCA AAG ATG GCT AGG‐3′ R: 5′‐CAG GAA CTG GAT CAG GAC TTT‐3′
	IL‐13	F: 5′‐GGT CAA CAT CAC CCA GAA CC‐3′ R: 5′‐TTT ACA AAC TGG GCC ACC TC‐3′
	TNF‐α	F: 5′‐CCC GAG TGA CAA GCC TGT AG‐3′ R: 5′‐GAT GGC AGA GAG GAG GTT GAC‐3′
	GAPDH	F: 5′‐CGT CTT CAC CAC CAT GGA GA‐3′ R: 5′‐CGG CCA TCA CGC CAC AGT TT‐3′

### 2.8. Induction of AD‐Like Skin Lesions in NC/Nga Mice and ATOJIN Treatment

DNCB is commonly used in AD research with NC/Nga mice because it induces chronic skin inflammation [21]. After a week of acclimatization, the dorsal hair of all NC/Nga mice was removed using an electronic clipper and hair removal cream prior to inducing AD‐like skin lesions with DNCB (Sigma Aldrich, Saint Louis, MO, USA) treatment. Specifically, 200 μL of 1% DNCB, dissolved in a 2:3 (v/v) mixture of acetone and olive oil, was applied to both ears and the dorsal skin on 0 and 3 days to induce sensitization. This was followed by the application of 200 μL of 0.4% DNCB, dissolved in a 3:1 (v/v) mixture of acetone and olive oil, to the same areas on Days 7 and 11 for the challenge. After numbering the NC/Nga mice, the numbers were drawn from a sealed, opaque envelope and randomly assigned to six groups (*n* = 7): a nontreated control group (standard naïve control), a DNCB‐treated group receiving PBS (negative control), a DNCB‐treated group receiving 0.1% tacrolimus (positive control), and DNCB‐treated groups receiving ATOJIN at doses of 400, 600, or 800 mg/kg. Mice in the negative control (PBS) and treatment groups (ATOJIN 400, 600, or 800 mg/kg) were administered the treatments orally on a daily basis for 2 weeks, while positive control mice received 0.1% tacrolimus twice weekly for the same duration. Ear thickness was measured using an electronic digital caliper (Mitutoyo, Tokyo, Japan). On Day 28, mice were anesthetized with Avertin (2,2,2‐tribromoethanol, 100 mg/kg, intraperitoneal injection) and euthanized by cervical dislocation under deep anesthesia. No animals were excluded from this study. The investigators collected and analyzed the data while blinded to group allocation, with samples processed in a randomized order. Histological analysis, white blood cell analysis in peripheral blood, and measurements of IgE and cytokine levels were conducted on samples collected on Day 28.

### 2.9. Assessment of Body Weight and Spleen Index

During the experimental period, the body weight of the mice was measured twice weekly. On the final day of the experiment, body weight was recorded before euthanasia, followed by the measurement of spleen weight. The spleen index (spleen weight [mg]/body weight [g]) was calculated to reflect the physiological changes and immune responses of the mice.

### 2.10. Evaluation of Skin Lesions

The severity of the dermatitis was assessed twice a week using the SCORing AD (SCORAD) index [27]. The severity of erythema/hemorrhage, edema, scarring/dryness, and excoriation/erosion was scored as follows: 0 (none), 1 (mild), 2 (moderate), and 3 (severe). The dermatitis score, which has a maximum of 12, was calculated as the sum of the individual scores and independently evaluated by three trained investigators who were blinded to the experimental groups.

### 2.11. Histological Analysis

Dorsal skin and ears were harvested from the mice for histological analysis on Day 28. The tissues were fixed in a 4% formalin solution for 24 h and subsequently embedded in Tissue‐Tek optical cutting temperature (OCT) compound (Leica Biosystems, Nussloch, Germany). Sections with a thickness of 10 μm were obtained using a microtome, mounted on glass slides, and dried overnight at 37°C. The tissue sections were stained with hematoxylin and eosin (H&E) to visualize inflammatory cells and with toluidine blue (TB) to identify mast cells. The slides were examined and photographed under a high‐power light microscope (Axio Scope A1, ZEISS, Oberkochen, Germany). Mast and inflammatory cells were counted in three high‐power fields (HPFs) at 200x and 400x magnification.

### 2.12. Analysis of White Blood Cells in Peripheral Blood

For the analysis of white blood cells, including neutrophils, eosinophils, basophils, monocytes and lymphocytes, and blood samples were placed in evacuated tubes containing K2‐EDTA as an anticoagulant (Becton Dickinson, Franklin Lakes, NJ, USA). The hematological parameters were then analyzed using flow cytometry at Biotoxtech Co., Ltd. (Ochang, Korea).

### 2.13. Measurement of IgE and Cytokine Levels by ELISA

Blood samples were collected from the mice via cardiac puncture under deep anesthesia induced by Avertin (100 mg/kg, intraperitoneal injection). Adequate depth of anesthesia was confirmed by the absence of a pedal withdrawal reflex before cardiac puncture. Serum was separated by centrifugation at 13,000 rpm for 1 min. The resulting serum samples were stored at −80°C until further use. Total levels of IgE and tumor necrosis factor (TNF)‐α were measured according to the manufacturer’s protocol using mouse ELISA kits from R&D Systems, Inc. (MN, USA).

### 2.14. Statistical Analysis

All data were analyzed using GraphPad Prism version 8.0 software (GraphPad Software, Inc.). One‐way ANOVA was employed, followed by Dunnett’s test to compare group differences against the control groups. For analyses requiring comparisons across multiple factors, two‐way ANOVA was used, followed by Tukey’s multiple comparisons to evaluate group differences. Results are presented as means and standard deviations (SDs). Statistical significance (*p*‐value) was defined as follows: # *p*  < 0.05, ^##^ *p*  < 0.01, and ^###^ *p*  < 0.001 compared to the nontreated control group (standard naïve control, Normal); *p*  < 0.05,  ^∗^ *p*  < 0.01, and  ^∗∗∗^ *p*  < 0.001 compared to the DNCB‐treated group receiving PBS (negative control, PBS). The animal experiment was conducted as a single trial.

## 3. Results

### 3.1. HPLC Analysis of ATOJIN

Qualitative and quantitative analyses were conducted to identify and quantitatively assess the active compounds in ATOJIN by comparing them with standard compounds. Figure 1A presents the content analysis of the expected standard compounds in the components of ATOJIN. As a result, Berberine, Oxymatrine, and Glycyrrhizin were identified as the marker compounds for PAR, SF, and GU, respectively (Figure 1B–D). However, the marker compounds for ST and LP were detected only at trace levels.

Figure 1HPLC profile of ATOJIN. (A) Composition of ATOJIN, showing the contents of individual plant extracts—*Phellodendron amurense Ruprecht* (PAR), *Schizonepeta tenuifolia* (ST), *Sophora flavescens* (SF), *Glycyrrhiza uralensis* (GU), and *Liriope platyphylla* (LP)—and the amount of standard compounds. (B–D) HPLC chromatograms showing the identification of standard compounds (berberine, oxymatrine, and glycyrrhizin) compared to ATOJIN.

**Figure:**
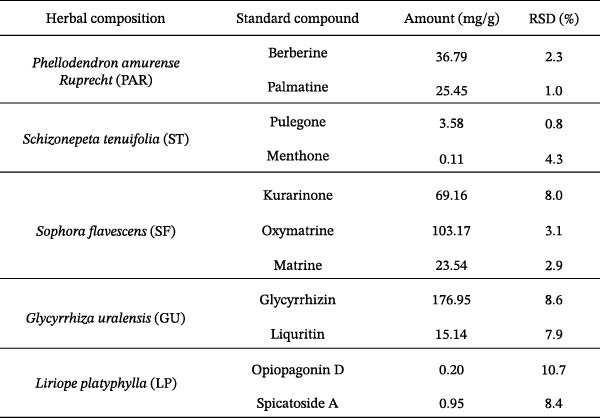
(A)

**Figure:**
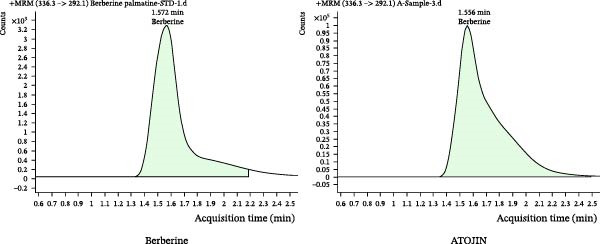
(B)

**Figure:**
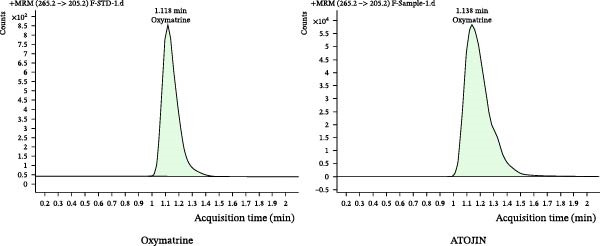
(C)

**Figure:**
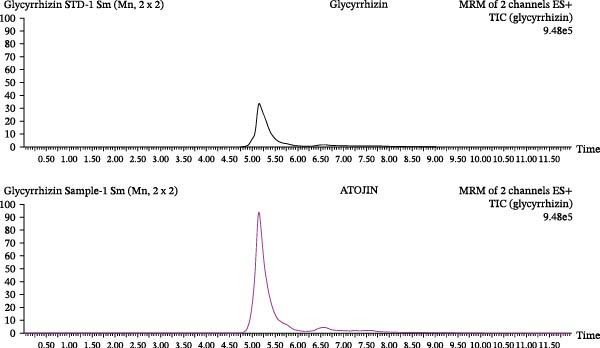
(D)

### 3.2. Effects of ATOJIN on DNCB‐Induced AD‐Like Symptoms in NC/Nga Mice

A DNCB‐induced AD mouse model was used to investigate the *in vivo* effects of ATOJIN on AD‐like skin inflammation. The experimental procedure is summarized in Figure 2A. Treatment with ATOJIN or tacrolimus alleviated AD‐like skin lesions. Data from Figure 2B,C show the monitoring of dermatitis scores and ear thickness throughout the experimental period. Additionally, the weight of the mice was continuously monitored, revealing no significant changes, which suggests that ATOJIN did not exhibit toxic effects (Figure 2D). As indicated in Figure 2E,F, the groups treated with ATOJIN demonstrated a reduction in dermatitis scores and ear dermal thickness compared to the control group. The spleen index was measured to assess immune status and was found to be increased in DNCB‐induced mice; however, ATOJIN treatment normalized this index (Figure 2G). In conclusion, ATOJIN treatment alleviated DNCB‐induced AD‐like skin lesions, and its efficacy was comparable to, or greater than, that of tacrolimus, a commonly used treatment for AD.

Figure 2Effects of ATOJIN on clinical symptoms in DNCB‐induced NC/Nga mice. (A) Schematic diagram of the study protocol. Clinical features of NC/Nga mice, including (B) dermatitis score, (C) ear thickness, and (D) body weight, were evaluated twice a week throughout the experimental period. *n* = 7 per group. Points and bars indicate the mean and standard deviation (SD) for each group of mice, respectively. *p*‐values were calculated using a two‐way ANOVA test (ns, not significant). At the end of the experiment (Day 28), data were extracted to highlight group differences: (E) dermatitis score, (F) ear thickness, and (G) spleen index, which was calculated as the ratio of spleen weight to body weight. *n* = 7 per group. *p*‐Values were calculated using one‐way ANOVA (ns, not significant; ^###^ *p* < 0.001 compared to the non‐treated control group;  ^∗^ *p* < 0.05,  ^∗∗^ *p* < 0.01, and  ^∗∗∗^ *p* < 0.001 compared to the DNCB‐treated group receiving PBS).

**Figure:**
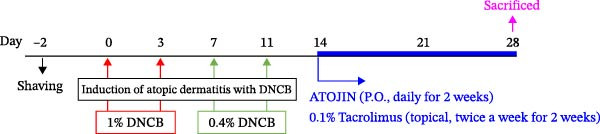
(A)

**Figure:**
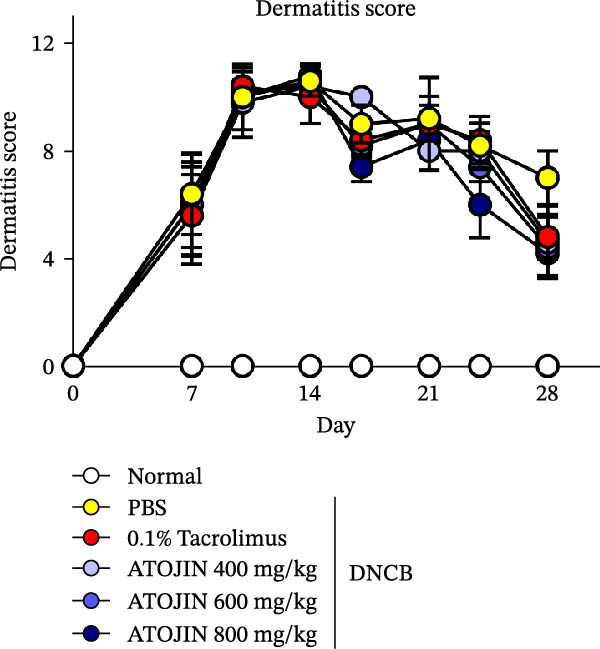
(B)

**Figure:**
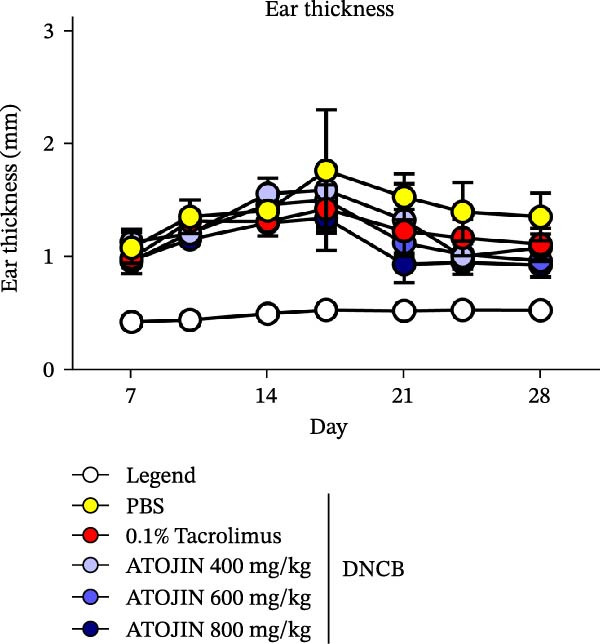
(C)

**Figure:**
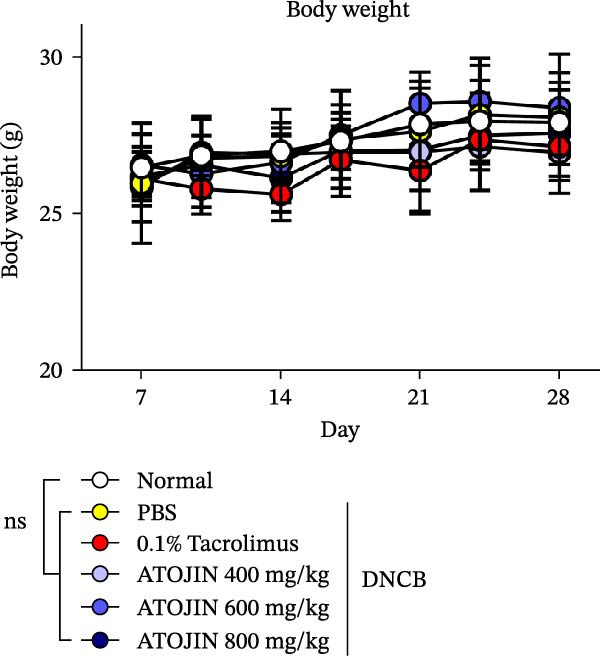
(D)

**Figure:**
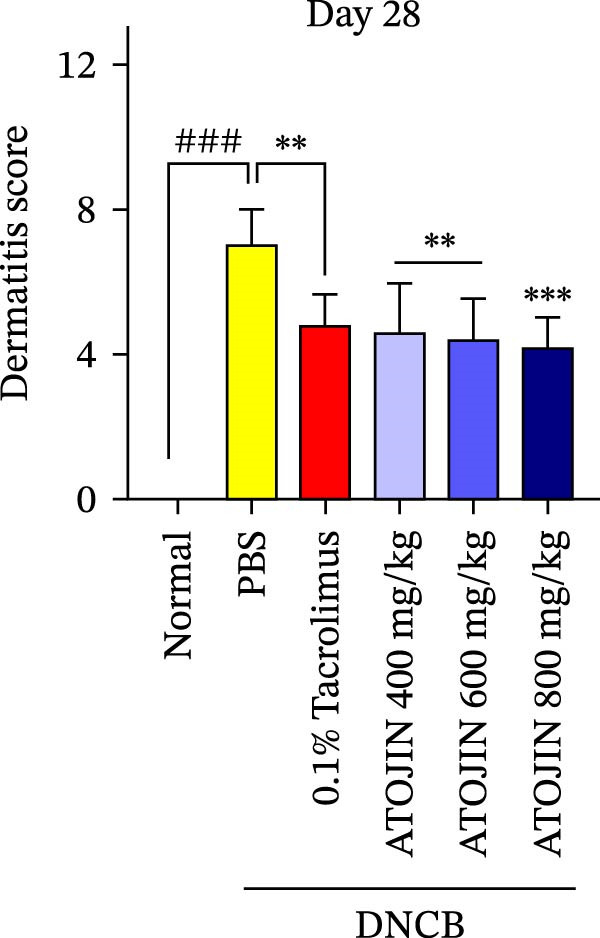
(E)

**Figure:**
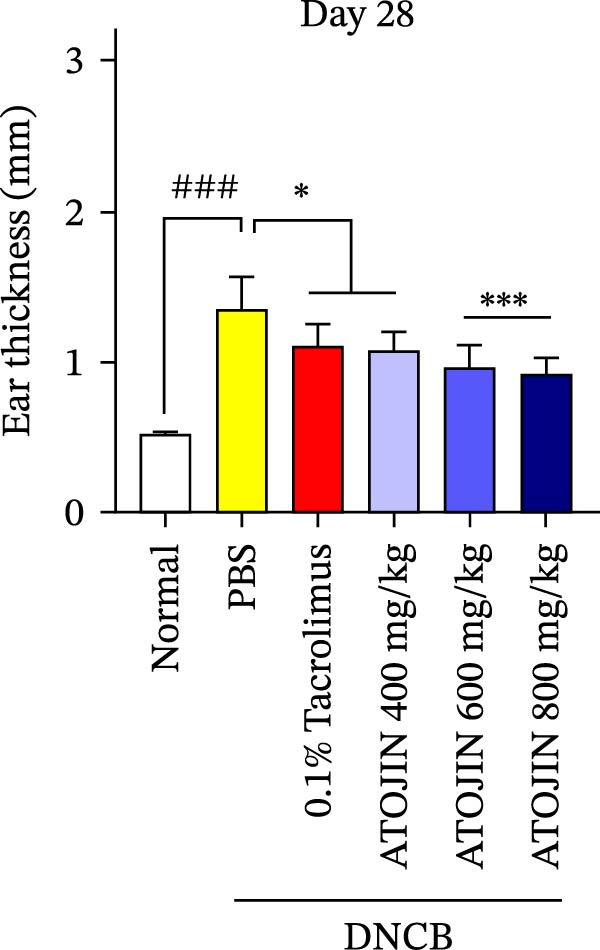
(F)

**Figure:**
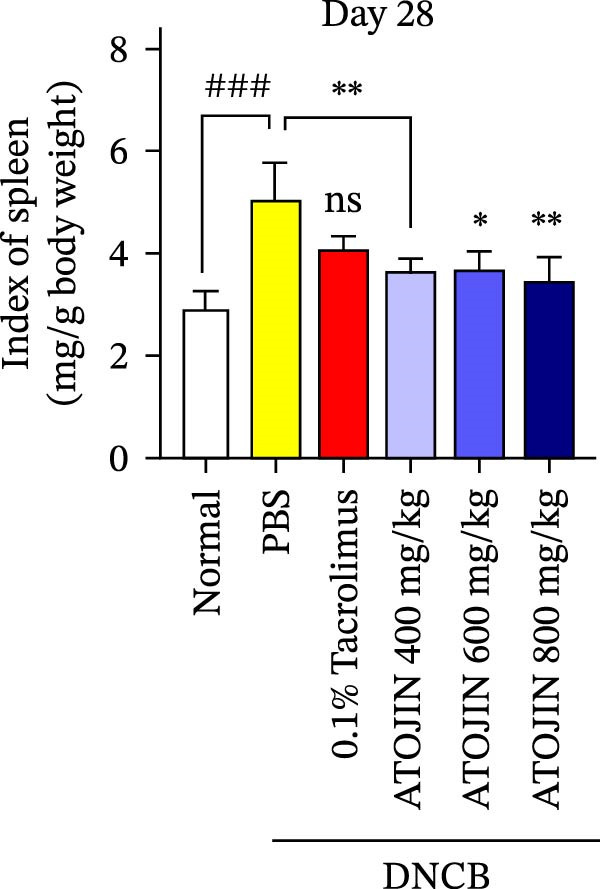
(G)

### 3.3. ATOJIN Effectively Inhibited the Thickening of the Epidermis and the Infiltration of Inflammatory Cells and Mast Cells

H&E staining was performed to assess inflammatory cell recruitment and dermal thickness in the DNCB‐induced mouse model. Dermal thickness and inflammatory cell infiltration were higher in the control group compared to the standard group but were significantly reduced in the tacrolimus and ATOJIN treatment groups (Figure 3A–C). Notably, in the ATOJIN treatment groups, dermal thickness decreased in a dose‐dependent manner, reaching levels comparable to those in the standard group. TB staining was conducted to evaluate mast cell infiltration, which was markedly increased in the control group compared to the standard group. Treatment with ATOJIN dose‐dependently reduced the number of mast cells infiltrating the ears and back of DNCB‐stimulated mice (Figure 3D,E).

Figure 3Effects of ATOJIN on the histopathological changes of skin lesions. (A) Skin biopsies stained with hematoxylin and eosin (H&E) reveal changes in the epidermis, as observed under a bright‐field microscope at 200x and 400x magnifications. *n* = 7 per group. (B) Epidermal thickness [black line in (A)] was measured in the H&E‐stained sections. *n* = 7 per group. (C) Inflammatory cells are counted in the H&E‐stained section by purple spots [blue arrows in (A)] and shown in a graph. *n* = 7 per group. (D) Skin biopsies stained with toluidine blue reveal mast cells, indicated by purple spots (black arrows), under a bright‐field microscope at 200x and 400x magnifications. *n* = 7 per group. (E) Mast cells were quantified, and the results are presented in a graph. *n* = 7 per group. Bars indicate the mean and standard deviation (SD) for each group of mice, respectively. *p*‐Values were calculated using one‐way ANOVA (^#^ *p*  < 0.05, ^##^ *p*  < 0.01, and ^###^ *p*  < 0.001 compared to the non‐treated control group;  ^∗^ *p* < 0.05,  ^∗∗^ *p* < 0.01, and  ^∗∗∗^ *p* < 0.001 compared to the DNCB‐treated group receiving PBS).

**Figure:**
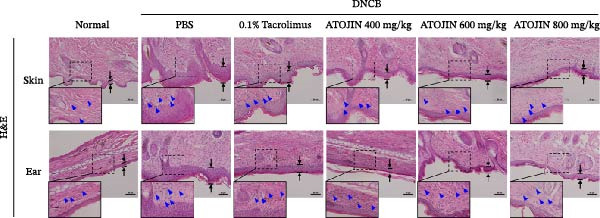
(A)

**Figure:**
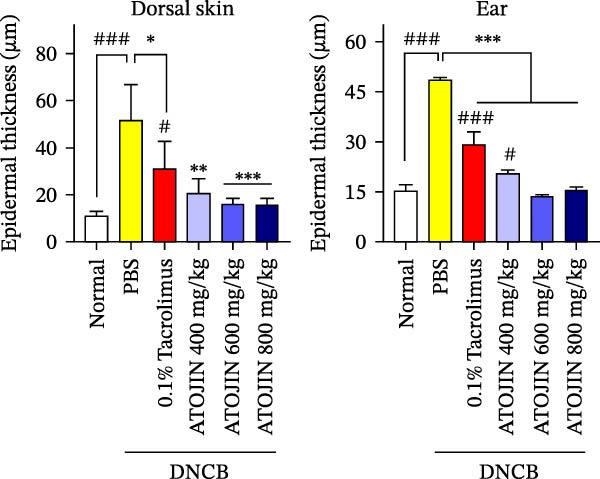
(B)

**Figure:**
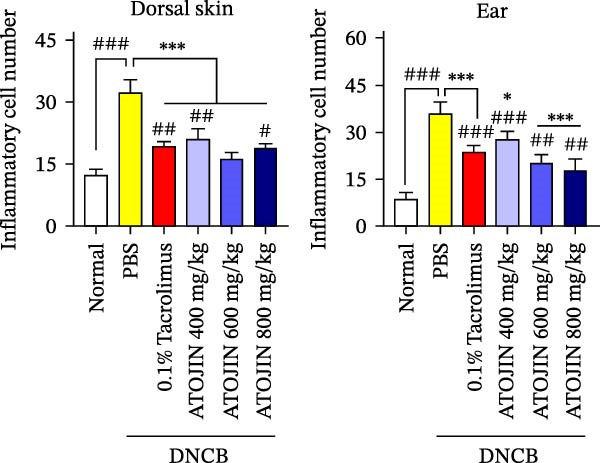
(C)

**Figure:**
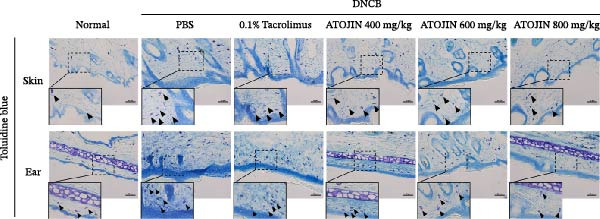
(D)

**Figure:**
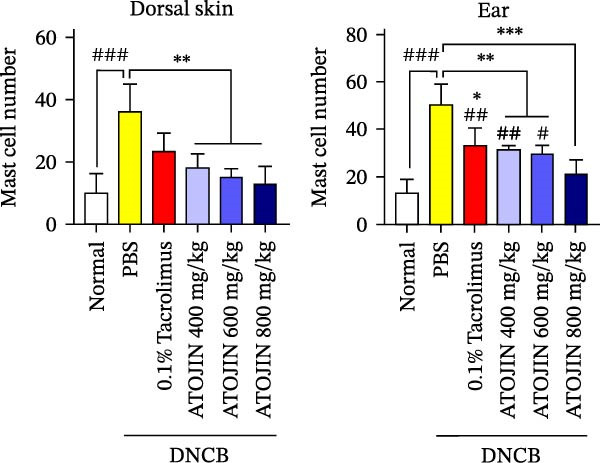
(E)

### 3.4. ATOJIN Regulates mRNA Expression of Inflammatory Cytokines in the Skin

**Figure 4:**
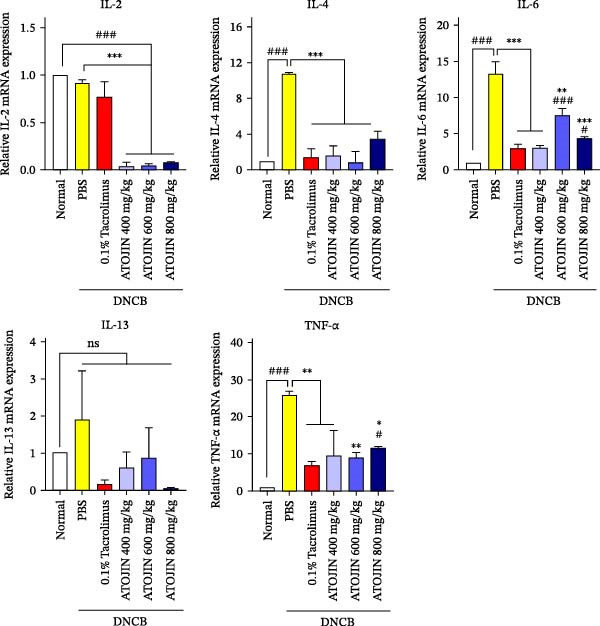
Effects of ATOJIN on mRNA expression of inflammatory cytokines in skin. mRNA expression levels of IL‐2, IL‐4, IL‐6, IL‐13, and TNF‐α in mouse skin tissue were measured by RT‐PCR. *n* = 7 per group. Bars indicate the mean and standard deviation (SD) for each group of mice, respectively. *p*‐Values were calculated using one‐way ANOVA (ns, insignificant; ^#^ *p*  < 0.05 and ^###^ *p*  < 0.001 compared to the non‐treated control group;  ^∗^ *p* < 0.05,  ^∗∗^ *p* < 0.01, and  ^∗∗∗^ *p* < 0.001 compared to the DNCB‐treated group receiving PBS).

To investigate the levels of cytokines secreted by Th1/Th2 cells in the ear skin, we measured the mRNA expression of interleukin (IL)‐2, IL‐4, IL‐6, IL‐13, and TNF‐α. The results indicated that mRNA levels of IL‐4, IL‐6, and TNF‐α were significantly increased in the skin lesions of the PBS + DNCB group compared to the normal control group, while no significant changes were observed in the levels of IL‐2 or IL‐13. Treatment with ATOJIN or tacrolimus significantly reduced the expression of IL‐4, IL‐6, and TNF‐α (Figure 4). Notably, IL‐2, which activates lymphocytes and induces the production of various inflammatory cytokines [28], was significantly reduced by ATOJIN treatment compared to tacrolimus, with levels lower than those in the control group. These results suggest that ATOJIN may modulate the expression of AD‐associated inflammatory cytokines in the skin, alleviating the immune response imbalance and positioning it as a potential therapeutic agent for modulating the immune response in AD.

### 3.5. ATOJIN Reduces the Number of WBCs in the Peripheral Blood of Mice

Since most WBC types tend to be elevated in patients with AD [6, 29], this study measured WBC counts and lymphocyte levels in the peripheral blood of mice. The results of the blood analysis indicated that both ATOJIN and tacrolimus inhibited the increase in WBC counts induced by DNCB (Figure 5A).

Figure 5ATOJIN regulated serum IgE, TNF‐α levels, and peripheral blood cell populations. On the final day of the experiment, blood samples were collected by cardiac puncture. (A) Hematological cell populations were analyzed using flow cytometry (Biotoxtech Co., Ltd., Ochang, Korea), and (B) IgE and TNF‐α serum levels were measured using ELISA kits. *n* = 7 per group. Bars indicate the mean and standard deviation (SD) for each group of mice, respectively. *p*‐Values were calculated using one‐way ANOVA (ns, insignificant; ^#^ *p*  < 0.05, ^##^ *p*  < 0.01, and ^###^ *p*  < 0.001 compared to the non‐treated control group;  ^∗^ *p* < 0.05,  ^∗∗^ *p* < 0.01, and  ^∗∗∗^ *p* < 0.001 compared to the DNCB‐treated group receiving PBS).

**Figure:**
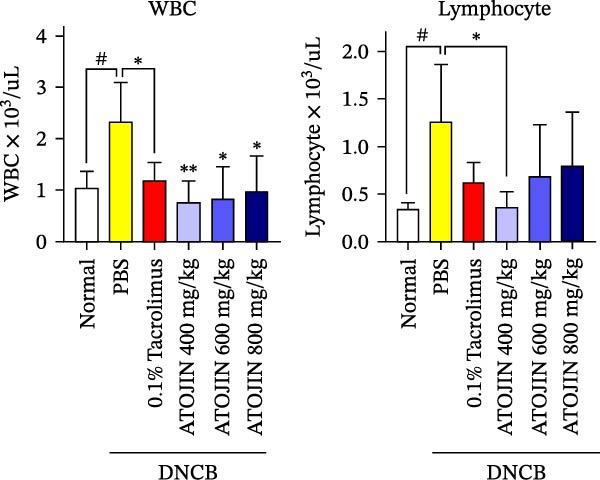
(A)

**Figure:**
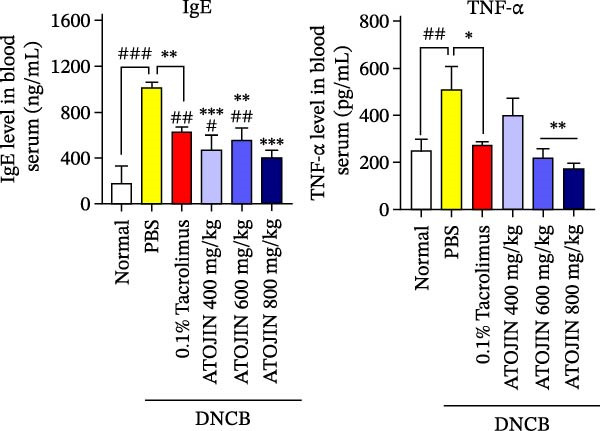
(B)

### 3.6. ATOJIN Reduces the Levels of IgE and TNF‐α in Mice Serum

TNF‐α is a critical cytokine that activates and recruits inflammatory cells, promoting inflammatory responses and worsening skin inflammation [30–32]. IgE plays a crucial role in allergic reactions, and its levels are utilized to diagnose and monitor AD [33]. To evaluate the effects of ATOJIN, we measured the levels of IgE and TNF‐α in mouse serum. ATOJIN reduced serum IgE and TNF‐α levels elevated by DNCB, demonstrating effects similar to those of tacrolimus. Notably, IgE levels were reduced to nearly normal levels, more effectively than with tacrolimus. (Figure 5B).

## 4. Discussion

This study demonstrates that oral administration of ATOJIN effectively alleviates the systemic symptoms of AD. AD is a chronic inflammatory skin disease that is becoming increasingly prevalent among children worldwide. Current treatments for AD, such as antihistamines, steroids, and immunosuppressants, have limited efficacy and are associated with significant side effects when used long‐term [34, 35]. There is a growing interest in treatments derived from natural products, including herbs, plants, mushrooms, yeasts, and flowers. Ongoing research is focused on exploring their biologically active components [36–38]. Currently, various natural products are being evaluated as potential therapeutic options due to their promising efficacy and relatively low risk of side effects, which expand their potential applications [37]. In this study, we propose ATOJIN, a treatment based on natural products for AD, and aim to elucidate its active compounds. AD medications are available in various forms, including topical applications and oral medications, each tailored to the disease’s progression and the patient’s condition. Topical applications provide rapid effects by acting directly on the affected area, although penetration may be incomplete. Additionally, prolonged use may diminish the skin’s resistance to infections [39, 40]. ATOJIN was administered orally to evaluate its potential as a systemic modulator of immune responses in AD, in contrast to topical agents like tacrolimus. By using systemic administration, ATOJIN may exert broader immunomodulatory effects, potentially alleviating AD symptoms beyond localized skin areas.

We first conducted a quantitative analysis of the standard compounds in the individual herbal extracts that make up ATOJIN. After examining the components and standardizing the raw materials, we identified berberine, oxymatrine, and glycyrrhizin as the primary active compounds in ATOJIN. These compounds have been investigated for their potential therapeutic effects in AD and conditions involving immune suppression [41–43]. The presence of these compounds indicates ATOJIN’s potential to positively influence multiple aspects of AD pathogenesis, thereby contributing to the alleviation of AD symptoms. Although the primary anti‐inflammatory compounds from ST and LP were selected based on previous reports [44–46], only trace levels were detected in our analysis. This suggests that ST and LP may enhance ATOJIN’s overall efficacy through additional bioactive constituents that have yet to be identified. Moreover, the greater anti‐inflammatory efficacy observed in the multi‐herbal formulation compared to the individual extracts implies that ATOJIN’s therapeutic effects may result from synergistic or additive interactions among its components, rather than the dominant activity of a single compound or herb. Therefore, comprehensive phytochemical profiling and further studies are essential to identify these constituents and establish a more precise standardization strategy for ATOJIN.

The therapeutic effects of ATOJIN observed in DNCB‐induced NC/Nga mice—such as reductions in dermatitis scores, ear thickness, and normalization of the spleen index—align with findings reported for other anti‐atopic agents that exhibit anti‐inflammatory and immunomodulatory potential in AD models [21, 47]. These results suggest that ATOJIN may modulate both local skin inflammation and systemic immune responses, highlighting its potential as a natural therapeutic option for AD. Importantly, no signs of toxicity were detected, confirming the preclinical safety of ATOJIN. Additionally, we established the safety of ATOJIN in vitro, showing lower cytotoxicity compared to its individual herbal components. At noncytotoxic concentrations, ATOJIN significantly reduced the expression of pro‐inflammatory cytokines in cellular models (Supporting Information: Figure S1), providing preliminary evidence of its anti‐inflammatory action at the cellular level.

Thickening of the skin (hyperkeratosis) is a recognized symptom of AD [48]. Histological analysis has shown that ATOJIN contributes to a reduction in epidermal thickness, as well as decreases in mast cell and inflammatory cell infiltration (Figure 3). This suggests that ATOJIN may help mitigate keratinization in AD. Based on these findings, we aimed to evaluate whether ATOJIN could restore immune balance by influencing the expression of cytokines associated with AD.

The clinical severity of AD is strongly correlated with increased circulating IgE produced by B lymphocytes and excessive inflammatory cytokine production by Th1 and Th2 lymphocytes [1–3]. Elevated IgE levels trigger both acute and chronic inflammation [49, 50]. Additionally, increased secretion of IL‐4, IL‐6, IL‐13, and TNF‐α by Th2 cells exacerbates immune imbalance and accelerates the progression of AD [51, 52]. Notably, IL‐2 produced by Th1 cells enhances T cell proliferation and activation, leading to increased release of inflammatory cytokines and a more intense inflammatory response in AD.

Our data indicate that ATOJIN decreases levels of inflammatory cytokines, including IL‐2, IL‐4, IL‐6, and TNF‐α in skin tissue (Figure 4), and lowers serum IgE levels (Figure 5B), demonstrating both systemic and localized anti‐inflammatory effects. Notably, ATOJIN significantly reduced IL‐2 levels compared to the control group, which aligns with the observed decrease in lymphocytes and total WBCs in the peripheral blood of mice. Consequently, ATOJIN helps modulate immune responses and reduce inflammation in AD. The suppression of IL‐2, IL‐4, IL‐6, and TNF‐α is generally associated with the inhibition of upstream signaling pathways such as NF‐κB, MAPK, and JAK‐STAT [53–55]. Therefore, it is plausible that ATOJIN may modulate these pathways to exert its anti‐inflammatory effects. Several key components of ATOJIN, including berberine, oxymatrine, and glycyrrhizin, have been reported to modulate inflammatory pathways in various models [56–58]. This suggests that ATOJIN may exert its anti‐inflammatory effects through similar mechanisms. To address safety concerns regarding ATOJIN, we administered it at various doses (400, 600, and 800 mg/kg) to evaluate its anti‐atopic efficacy. Our data revealed that the 400 mg/kg dose achieved therapeutic efficacy comparable to tacrolimus across multiple parameters, indicating that this dose may already be sufficient for effective treatment.

To enhance its clinical applicability, future studies should aim to determine the minimal effective dose of ATOJIN, identify its specific anti‐inflammatory bioactive constituents and their underlying molecular mechanisms, and conduct a comprehensive assessment of its safety and pharmacological profile under both pathological and normal conditions.

## 5. Conclusions

This study evaluated the therapeutic effect of ATOJIN in a model representing the moderate inflammation stages of AD. Our research indicates that ATOJIN administration significantly alleviates AD symptoms. Notably, ATOJIN has demonstrated comparable or superior effectiveness in anti‐atopic effects compared to tacrolimus, a commonly prescribed AD medication. Therefore, ATOJIN may serve as a novel therapeutic option for AD, particularly during the maintenance phase. However, further studies are needed to assess its safety and molecular mechanisms, determine the optimal therapeutic dose, and confirm its clinical efficacy during both maintenance and active phases of inflammation to enhance its potential applicability.

## Author Contributions

**Sang-Eun Lee and Kwang-Jin Cho:** data curation, investigation, validation, writing – original draft. **Min-Woo Kim:** data curation, investigation, validation. **Hee-Sun Yim:** conceptualization, funding acquisition, project administration, resources, writing – review and editing. **Seong-Gyu Ko:** conceptualization, project administration, supervision, funding acquisition, writing – review and editing.

## Funding

This work was supported by the National Research Foundation of Korea (NRF) grant funded by the Korea Government (MSIT) (Grant RS‐2020‐NR049559) and by the Starting Growth Technological R&D Program (Grant RS‐2023‐00265218) funded by the Ministry of SMEs and Startups (MSS, Korea).

## Disclosure

After using ChatGPT‐5.2, OpenAI tool, the authors reviewed and edited the content as needed and took full responsibility for the content of the publication.

## Conflicts of Interest

The authors declare no conflicts of interest.

## Supporting Information

Additional supporting information can be found online in the Supporting Information section.

## Supporting information


**Supporting Information** Figure S1: Cytotoxicity and anti‐inflammatory effects of ATOJIN in vitro. (A) Cytotoxicity of ATOJIN and its individual herbal components in HDFα cells was evaluated using a WST assay. Cells were treated with increasing concentrations of ATOJIN (0–1000 μg/mL), and absorbance was measured to determine cell viability. (B) Cytotoxicity of ATOJIN and its individual herbal components in HMC‐1.1 cells stimulated with PMA (5 ng/mL) and ionomycin (500 ng/mL). (C) Real‐time PCR (RT‐PCR) analysis of pro‐inflammatory cytokine expression in HMC‐1.1 cells treated with noncytotoxic concentrations of ATOJIN (100 μg/mL). (ns, not significant; ^###^ *p* < 0.001 compared to the nontreated control group; *p* < 0.05,  ^∗^ *p* < 0.01, and  ^∗∗^ *p* < 0.001 compared to the negative control (PMA/ionomycin‐stimulated HMC‐1.1 treated with PBS); ^†††^ *p* < 0.001 compared to the negative control). All experiments were independently performed in triplicate. PAR, *Phellodendron amurense ruprecht*; ST, *Schizonepeta tenuifolia*; SF, *Sophora flavescens*; GU, *Glycyrrhiza uralensis*; LP, *Liriope platyphylla*.

## Data Availability

The data that support the findings of this study are available from the corresponding author upon reasonable request.
