# Supplementary figures and images for: ATOJIN: A Natural Products Mixture, Alleviates Atopic Dermatitis in DNCB‐Induced NC/Nga Mice
Source: Mediators Inflamm. 2026 Mar 14;2026:3444278. doi: 10.1155/mi/3444278 (PMC13140213; doi:10.1155/mi/3444278)

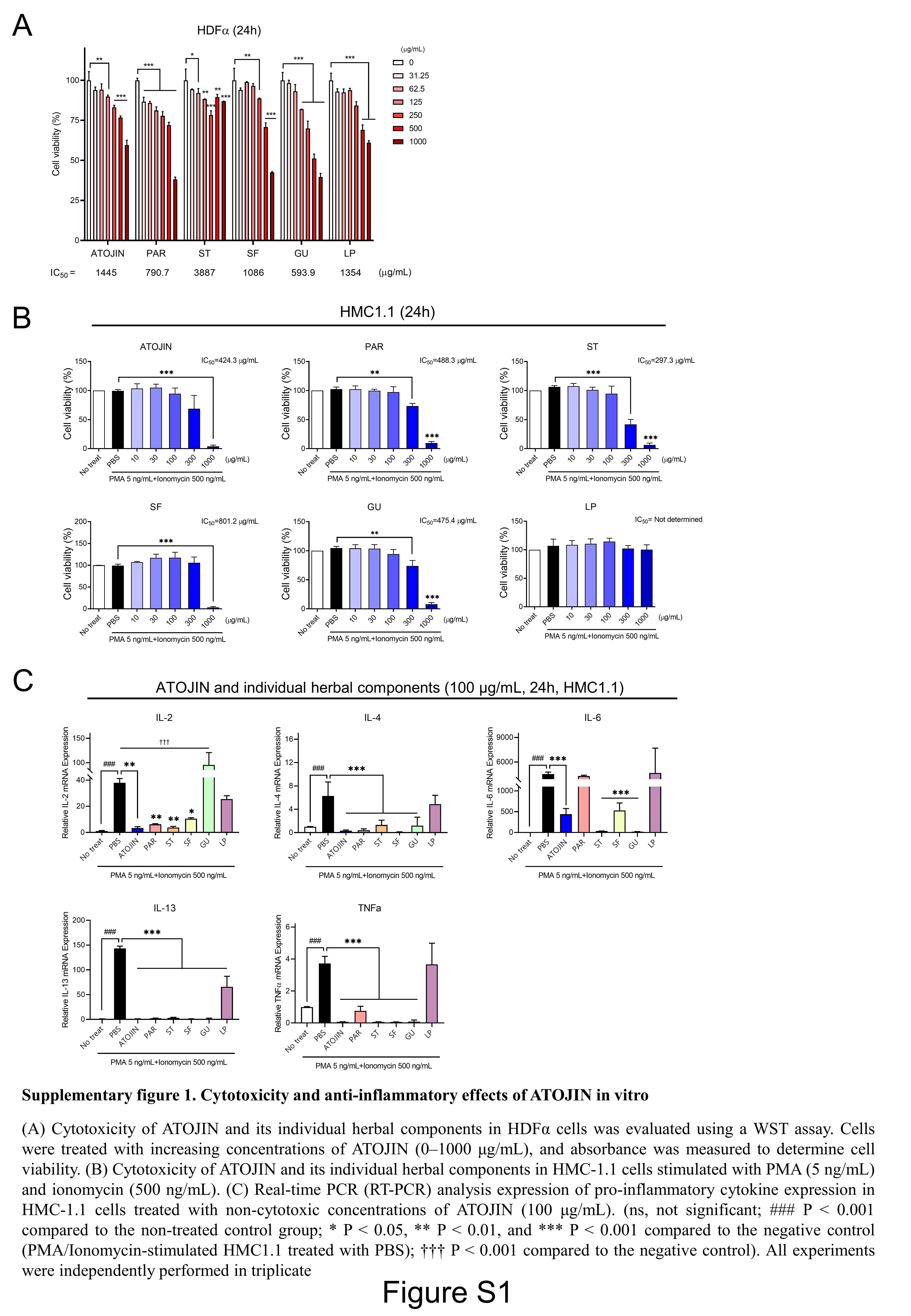

Supplement: Supplementary file 1 — Supporting Information Figure S1: Cytotoxicity and anti‐inflammatory effects of ATOJIN in vitro. (A) Cytotoxicity of ATOJIN and its individual herbal components in HDFα cells was evaluated using a WST assay. Cells were treated with increasing concentrations of ATOJIN (0–1000 μg/mL), and absorbance was measured to determine cell viability. (B) Cytotoxicity of ATOJIN and its individual herbal components in HMC‐1.1 cells stimulated with PMA (5 ng/mL) and ionomycin (500 ng/mL). (C) Real‐time PCR (RT‐PCR) analysis of pro‐inflammatory cytokine expression in HMC‐1.1 cells treated with noncytotoxic concentrations of ATOJIN (100 μg/mL). (ns, not significant; ### p < 0.001 compared to the nontreated control group; p < 0.05, ∗ p < 0.01, and ∗∗ p < 0.001 compared to the negative control (PMA/ionomycin‐stimulated HMC‐1.1 treated with PBS); ††† p < 0.001 compared to the negative control). All experiments were independently performed in triplicate. PAR, Phellodendron amurense ruprecht; ST, Schizonepeta tenuifolia; SF, Sophora flavescens; GU, Glycyrrhiza uralensis; LP, Liriope platyphylla. [file MI-2026-3444278-s001.tif]
